# Should Screening for Chlamydia Trachomatis Be Performed on Asymptomatic Young Adults as well as on the Victims of Sexual Assault?

**DOI:** 10.1089/whr.2020.0088

**Published:** 2021-01-12

**Authors:** Oriol Yuguero, Amaia Cabases, Silvia Bertran, Crisitina Molins, Paula Paredes, Maria Ramirez

**Affiliations:** ^1^ Transversal Emergency Research Group, Biomedical Research Institute of Lleida (IRBLLEIDA), Lleida, Spain.; ^2^ Catalan Health Institute, Health Region of Lleida, Lleida, Spain.; ^3^ Faculty of Medicine, University of Lleida, Lleida, Spain.

**Keywords:** chlamydia trachomatis, screening, sexual assault

## Abstract

In recent years, chlamydia trachomatis (CT) screening has been discussed among different scientific boards. However, in Spain, chlamydia screening is offered to women who attend a hospital after sexual assault. We found that 5.1% of 59 cases attending our hospital after sexual assault between January 2017 and December 2019 tested positive for chlamydia infection. The mean age of the cases was 23.3 years. In comparison, screening asymptomatic patients of similar age attending hospital for other reasons have revealed a prevalence of CT of 7%. Hence, since CT is common in asymptomatic individuals as well as victims of sexual assault, we believe that chlamydia screening should also be a priority in asymptomatic patients.

## Introduction

In recent years, chlamydia trachomatis (CT) infection has proved to be one of the most commonly reported sexually transmitted infections, as recently announced by the European Centre for Disease Prevention and Control (ECDC).^1^ According to data from the latest report by the Centre for Epidemiological Studies on Sexually Transmitted Diseases and HIV/AIDS of Catalonia (CEEISCAT),^2^ the global rate of CT in the entire population of Catalonia has increased from 6.6 cases per 100,000 inhabitants in 2011 to 93.5 cases in 2018, that is, a 14-fold increase among both men and women. Similar increments have been described in other regions and countries.^3^ In our hospital,^4^ we conducted a study of the prevalence of CT in patients attending a sexually transmitted diseases (STDs) unit that revealed a prevalence of CT of 9.3% in such patients.

To date, there are no studies evaluating the incidence of cases in patients who have suffered sexual assault in Spain, whereas such data have been published in other countries in Europe. CT infection is globally the most common bacterial STD, potentially causing serious complications in women's reproductive tract.^5^ In Catalonia, between 2017 and 2019, National Police Department reported that 1419 people were sexually assaulted (394 in 2017, 468 in 2018, and 557 in 2019).^6^ At all hospitals receiving patients for medical care after alleged sexual assault, a forensic examination is offered in case of later police report. All patients attending the emergency departments after a sexual assault are evaluated by an emergency doctor, a gynecologist, and a forensic specialist, who obtain forensic specimens.

## Methods and Results

Between January 2017 and December 2019, 59 sexual assault cases were treated by the emergency department of the Arnau de VIlanova University Hospital in Lleida. This hospital is a hospital of reference for an approximate rural population of 200,000.

All patients aged 16 years and above were included in the case review, of whom 93% were women and patient mean age was 23.3 years. Patients usually attend <12 hours after the assault. Only two patients had a previous STD infection.

At our hospital, sexually transmitted infection testing is offered to all patients who contact our hospital after sexual assault, in accordance with the ECDC recommendations.^7^

Upon screening, *CT, Mycoplasma Hominis, Trichomonas Vaginalis (TV),* and *Neisseria Gonorrhoeae* are detected by vaginal smears, and by anal smears if necessary using polymearse chain reaction/nucleic acid amplification tests. In addition, determination of herpes simplex virus, HIV, hepatitis B virus, hepatitis C virus, and syphilis is performed by serological testing. After screening, postexposure prophylactic STD and HIV treatment are administered. We administer ceftriaxone, metronidazole, and azithromycin to prevent STD, and raltegravir and truvada for HIV prophylactic treatment. We administer different drugs depending on renal filtrate and allergies. During the follow-up, the need for further treatment is evaluated. The purpose of this screening is to ascertain the patient's infection status. All patients are recommended a followed consultation at the regional infectious diseases service, a specific unit that receives patients with different infectious diseases and who are referred from primary health care or from other hospital units.

At the initial visit shortly after sexual assault, we found three cases (5.1%) testing positive for CT and four patients (6.7%) testing positive for TV. In a study conducted in France published in 2016, 6% tested positive for CT (and 6% for Mycoplasma genitalium), which justifies performing STD testing among patients attending health care shortly after sexual assault.^8,9^ Similarly, in Norway, in a study published in 2014, the prevalence of CT infection in patients who suffered sexual assault was 6%; however, this was lower than in a comparable clinical population.^10^

All patients attending our hospital after sexual assault in the given period of this study were treated by prophylactic antibiotics. However, those testing positive for an STD unfortunately never came for a follow-up retest to check for eradication of their infection.

Our team is developing a screening program^11^ in the asymptomatic population of men and women between 18 and 25 years of age (preliminary data pending publication), and we found that the prevalence of CT infection was as high as 7.4% among this asymptomatic sample of young patients attending our emergency department due to other health problems. However, although recommended by the ECDC, screening in sexually active young patients between the ages of 15 and 25 years is not being carried out in our country.

This study was approved by the ethics research committee of the Biomedical Research Institute of Lleida.

## Conclusion

CT infection has increased in recent years,^1^ and its approach and prevention require the involvement of many health institutions and agencies. In the recent literature, there have been discussions as to the usefulness of immediate CT screening in patients who are the victims of sexual assault, although the incidence is close to 5%. In some countries, it is carried out at least 2 weeks after assault, and in Spain it is conducted on the same day to ascertain patient status at that moment, and the health system provides patients with a follow-up. With this screening, we pretend to meaure the baseline prevalence of pre-existing CT in the assault victims group.

In Spain, CT testing is performed at the same time as the forensic medical examination after recent sexual assault—suggesting that these patients are at a higher risk of, for example, CT or TV infection. By giving the women (and men) proper antibiotics to prevent serious infection after a possible assault-related transmission, the total burden in the aftermath of the assault may be reduced.

In Spain there is no screening program targeting the asymptomatic young population, where preliminary studies and experiences show an incidence >7% (7.4%) in asymptomatic subjects. It is for this reason that the implementation of a screening program in the asymptomatic sexually active young population should be considered, given the high prevalence and risks that this untreated infection can entail, especially among young people aged between 15 and 25 years.

We have seen that STD screening of young patients attending our local hospital either after being sexually assaulted or for any other reason could detect asymptomatic cases, and hence, potentially avoid complications of untreated STDs. Despite the relatively low prevalence among sexually assaulted cases, it is recommended to screen these patients for STDs. However, a screening program targeting young sexually active male and female patients with a possible higher STD positivity rate is still not recommended in our country.

## Abbreviations Used

CT: chlamydia trachomatis
ECDC: European Centre for Disease Prevention and Control
STD: sexually transmitted diseases
TV: trichomonas vaginalis
